# Development of a field testing protocol for identifying Deepwater Horizon oil spill residues trapped near Gulf of Mexico beaches

**DOI:** 10.1371/journal.pone.0190508

**Published:** 2018-01-12

**Authors:** Yuling Han, T. Prabhakar Clement

**Affiliations:** 1 Environmental Engineering Program, Department of Civil Engineering, Auburn University, Auburn, Alabama, United States of America; 2 Department of Civil, Construction and Environmental Engineering, University of Alabama, Tuscaloosa, Alabama, United States of America; University of California, Merced, UNITED STATES

## Abstract

The Deepwater Horizon (DWH) accident, one of the largest oil spills in U.S. history, contaminated several beaches located along the Gulf of Mexico (GOM) shoreline. The residues from the spill still continue to be deposited on some of these beaches. Methods to track and monitor the fate of these residues require approaches that can differentiate the DWH residues from other types of petroleum residues. This is because, historically, the crude oil released from sources such as natural seeps and anthropogenic discharges have also deposited other types of petroleum residues on GOM beaches. Therefore, identifying the origin of these residues is critical for developing effective management strategies for monitoring the long-term environmental impacts of the DWH oil spill. Advanced fingerprinting methods that are currently used for identifying the source of oil spill residues require detailed laboratory studies, which can be cost-prohibitive. Also, most agencies typically use untrained workers or volunteers to conduct shoreline monitoring surveys and these worker will not have access to advanced laboratory facilities. Furthermore, it is impractical to routinely fingerprint large volumes of samples that are collected after a major oil spill event, such as the DWH spill. In this study, we propose a simple field testing protocol that can identify DWH oil spill residues based on their unique physical characteristics. The robustness of the method is demonstrated by testing a variety of oil spill samples, and the results are verified by characterizing the samples using advanced chemical fingerprinting methods. The verification data show that the method yields results that are consistent with the results derived from advanced fingerprinting methods. The proposed protocol is a reliable, cost-effective, practical field approach for differentiating DWH residues from other types of petroleum residues.

## 1. Introduction

The Deepwater Horizon (DWH) accident, which resulted in one of the largest marine oil spills in U.S. history, released about 5.0 million barrels of crude oil into the Gulf of Mexico (GOM) [1]. Michel et al. [2] estimated that the DWH oil spill contaminated over 1,700 kilometers of GOM shoreline and impacted several amenity beaches, marshes and other ecologically sensitive coastal ecosystems. Along the Alabama shoreline, the spill impacted about 50 kilometers of sandy beaches located in between Orange Beach to Fort Morgan. These amenity beaches are major tourist attractions, and they support the local economy of several coastal towns. Therefore, there is considerable interest in understanding the post oil spill recovery levels, and also assessing the changes in background levels in the aftermath of the DWH oil spill. Field studies have shown that the DWH spill has substantially increased the background oil levels of several GOM beaches [3–5]. For example, based on multiple field datasets, Clement et al. [3] estimated that the background oil levels in Alabama’s beaches have increased by at least thousand fold. The average historic background level for Alabama’s beaches prior to the DWH oil spill event was estimated to be 2 g/km/year (about 2 to 4 tar balls/km/year; they are highly weathered tar balls with each weighing about 0.5 to 1 g). The levels estimated for Alabama beaches based on a field survey completed on January, 2016 ranged from 2,400 to 31,000 g/km/year [3]. Some of the beaches were heavily contaminated; for example, from a kilometer long beach in Fort Morgan they recovered 233 fragments of oil residues, weighting about 1,310 grams, within an hour [3]. The size of each residue ranged from 0.5 cm to 7 cm and the weight ranged from 0.5 to 50 g. Previous studies have also shown that the DWH oil spill residues found along GOM beaches have partially weathered crude oil that contain various types of toxic polycyclic aromatic hydrocarbons [5–8].

Historically, most GOM beaches are often contaminated by different types of petroleum residues (residues that are not of DWH origin) that could have originated from natural oil seeps, accidental releases from oil rigs and ship oil, or anthropogenic waste oil dumping activities [9,10]. Therefore, in order to assess the impacts of the DWH oil spill, one needs to identify and differentiate DWH residues from other petroleum residues. The DWH oil spill residues are often referred to as “tar balls,” a term that normally refers to rubbery, black, highly-weathered masses of oil, which could be found along some GOM beaches. However, DWH oil residues have several physical and chemical characteristics that are different from these traditional black tar balls [11].

When crude oil is discharged into the ocean, various weathering processes that are driven by winds and waves break the floating oil into smaller patches, which are then transported by ocean currents. During this process, almost all the lighter components in the crude oil rapidly evaporate [12]. The unevaporated portion of the oil eventually mixes with water to form a thick brownish emulsion, known as mousse [13]. When the mousse is stranded in deep ocean for a long period (several months to years), it will be stretched and torn apart by winds and waves to eventually form highly weathered black tar balls [14].

The DWH oil spill residues found along Alabama’s beaches were formed by different types of nearshore processes. The sweet crude oil released from the DWH well rapidly emulsified forming brownish mousse, and within weeks the mousse was transported by ocean currents towards the Alabama shoreline. When the floating weathered mousse approached the shoreline, it interacted with suspended sediments and sank in the nearshore environment. Gustitus and Clement recently presented a detailed conceptual model for describing the coastal transport mechanisms that facilitated the formation of sunken DWH oil spill residues [15]. After sinking, the submerged oil accumulated more sediments to form large deposits of oily mats, known as submerged oil mats or sediment-oil mats (SOMs). Waves and other shoreline transport processes eventually broke SOMs into smaller fragments, known as surface residual oil balls (SRBs) or sediment-oil agglomerates (SOAs) [15]. Typical size of SRB/SOAs found along the Alabama shoreline can range from about 0.5 cm to 8 cm [3,4]. Over time, these residues are physically disintegrated by nearshore erosion processes and become too small to be recoverable. Our field observations have indicated that SOAs less than about 0.5 cm are difficult to identify and hence are unlikely to be recovered.

Identifying the source of oil in various types of petroleum residues is an important task in any oil spill investigation [16–19]. Several published analytical procedures are available that can be used to objectively track and identify the source of oil spill residues. These procedures analyze recalcitrant biomarkers or other petroleum chemicals, which are naturally present in the crude oil, using advanced GC/FID or GC/MS methods [16,20,21]. After obtaining the analytical data, various methods are used to compute different types of ratios and fingerprints to differentiate the oils [18,22]. While these advanced source identification methods are highly efficient, it is rather impractical to routinely use them to analyze several hundreds (and even thousands) of field samples collected after a large spill event, such as the DWH oil spill [3,4]. Also, conducting advanced laboratory studies can be cost-prohibitive and almost infeasible for most local municipalities, regulatory agencies and community groups that use their own employees, temporary workers and/or volunteers to conduct the monitoring surveys. These field workers might not have sufficient training and/or have access to a laboratory to conduct detailed chemical characterization studies. Therefore, in order to develop cost effective long-term monitoring strategies, one would need simpler field methods that can be rapidly deployed and can used by minimally-trained field workers to identify oil spill residues. The focus of this study is to develop a practical field method for monitoring DWH oil spill residues that continue to be deposited along sandy GOM beaches in the form of SOM/SOAs.

As discussed before, DWH SOAs were formed by the rapid burial of emulsified mousse after it interacted with suspended sediments and sank in the nearshore environment. Our past laboratory studies and field observations have indicated that these conditions have resulted in forming oil residues that have distinctive physical characteristics [4,5,11]. The objective of this study is to develop a practical field method that can help assess some of these unique physical characteristics and use them to differentiate the DWH residues from other petroleum residues found along sandy GOM beaches. The robustness of the method was tested by analyzing several DWH residues along with other oil spill residues and comparing the results of the physical characterization method with the results obtained from detailed chemical characterization methods. This work is novel because, as per our knowledge, so far no one has developed a well-tested field protocol for identifying DWH oil spill residues based on a series of physical characterization tests.

## 2. Material and methods

### 2.1 Details of the field samples

A total of 11 oil spill samples were used in this study. Sample-1 is a well-known DWH reference sample, which was collected by our team during a field survey completed on September 24, 2011, at a site located about 500 meters west of Lagoon Pass in Gulf Shores, Alabama. This survey was a joint SOM excavation survey completed by the U.S. Coast Guard, Alabama Department of Environmental Management and Auburn University. Sample 1 is considered as a DWH reference sample since it has been extensively analyzed in other published work [23].

**Fig 1:**
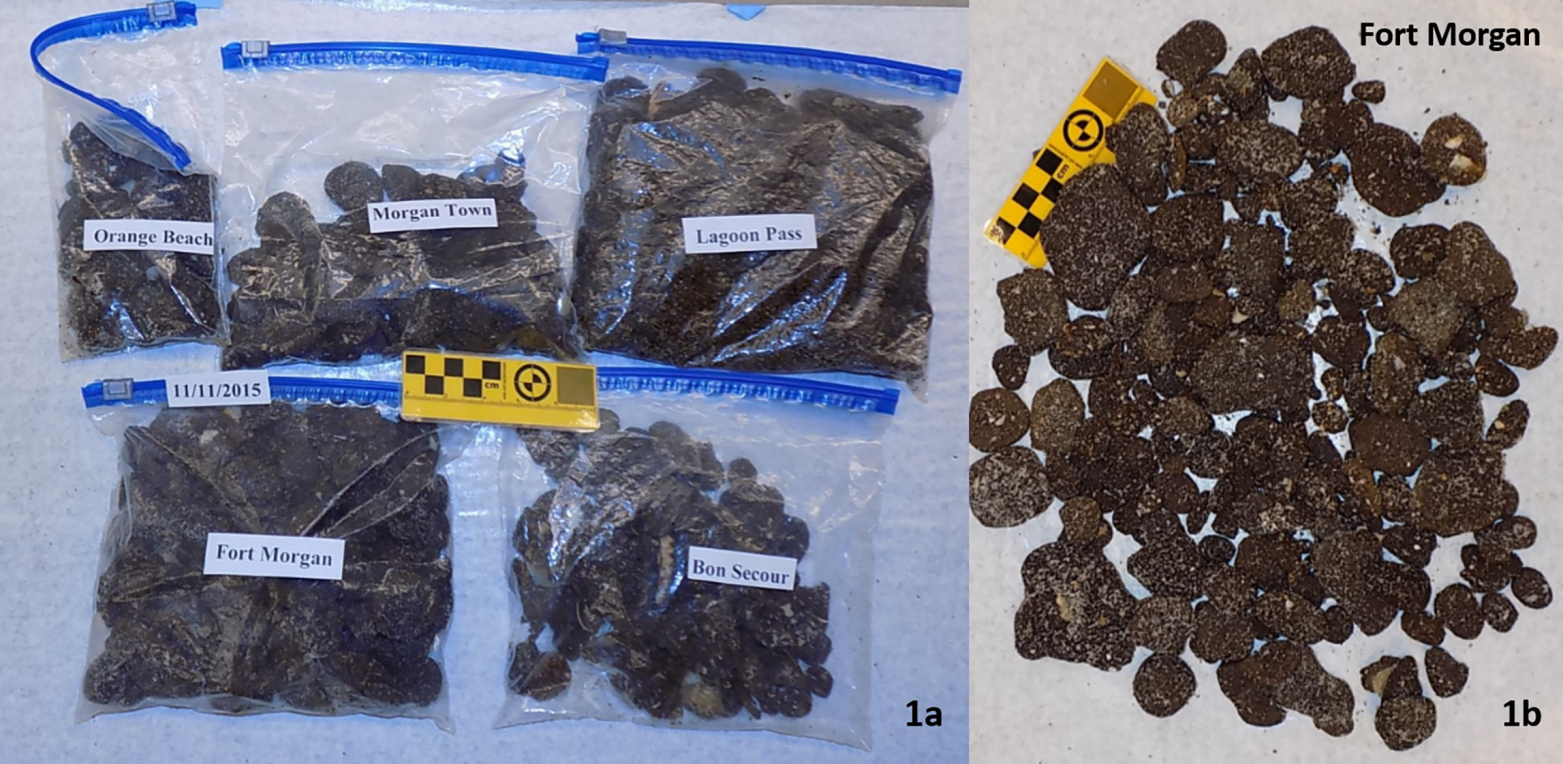
Oil spill residues collected from five different Alabama beaches on November 11th 2015 in the left side picture and residues collected from Fort Morgan (218 samples of total weight 836 grams; sampling distance ~1 km, and sampling time ~1 hour) in the right side picture.

Samples 2 through 6 were collected during a detailed beach survey which was completed by our team along the Alabama shoreline on November 11, 2015. During this survey, we collected 693 pieces of oil residues, with a total weight of 2,376 g, from five beaches located in between Fort Morgan and Orange Beach. Fig 1A (left) shows all the samples collected during this survey, and Fig 1B (right) shows a close-up view of samples collected from Fort Morgan beach (beach length ~1 km) facing the Mobile Bay. Table 1 provides a summary of the size distribution of the residues collected during this survey. Sizes of the samples were determined by measuring the length (largest dimension) using a ruler. Samples 2 through 6 were random samples selected from the residues collected at Lagoon Pass (30°14'24.0"N 87°44'24.0"W), Fort Morgan (30°13'52.1"N 88°01'15.2"W), Morgan Town (30°13'50.38"N 87°54'33.69"W), Bon Secour (30°13′44.90″N 87°49′41.26″W) and Orange Beach (30°16'18.6"N 87°34'18.0"W), respectively.

**Table 1:** Details of DWH oil spill residues collected during a field survey completed on Nov. 11, 2015.

Location	Weight (g)	Total numbers	Size Distribution (cm)				
			~1	~2	2–3	3–4	4–7
Lagoon Pass	685	108	84	47	25	19	5
Fort Morgan	836	218	102	61	17	29	9
Morgan Town	373	107	35	46	13	12	1
Bon Secour	330	80	70	23	12	3	-
Orange Beach	152	180	32	34	6	8	-

Sample 7 was collected by our team from a beach in Goa, India (15°34'33.8"N 73°44'24.2"E), on September 15, 2014; tar balls are routinely deposited by southwest monsoon currents on several of Goa’s beaches [24]. Sample 8 was retrieved by our team from Gulfport, Mississippi (30°21'32.3"N 89°06'30.6"W) during a field survey completed in January 2013. Sample 9 was collected by our team in Grand Isle, Louisiana (29°14'11.8"N 89°59'14.3"W) during our field survey completed in January 2013. Sample 10, which looked like a traditional tar ball, was collected from Orange Beach, Alabama (30°16'18.6"N 87°34'18.0"W), during our November 2015 survey. Sample 11 was provided by the Texas General Land Office and was collected at a beach near Houston area (GPS coordinates are not available).

### 2.2 Details of the proposed protocol

**Fig 2:**
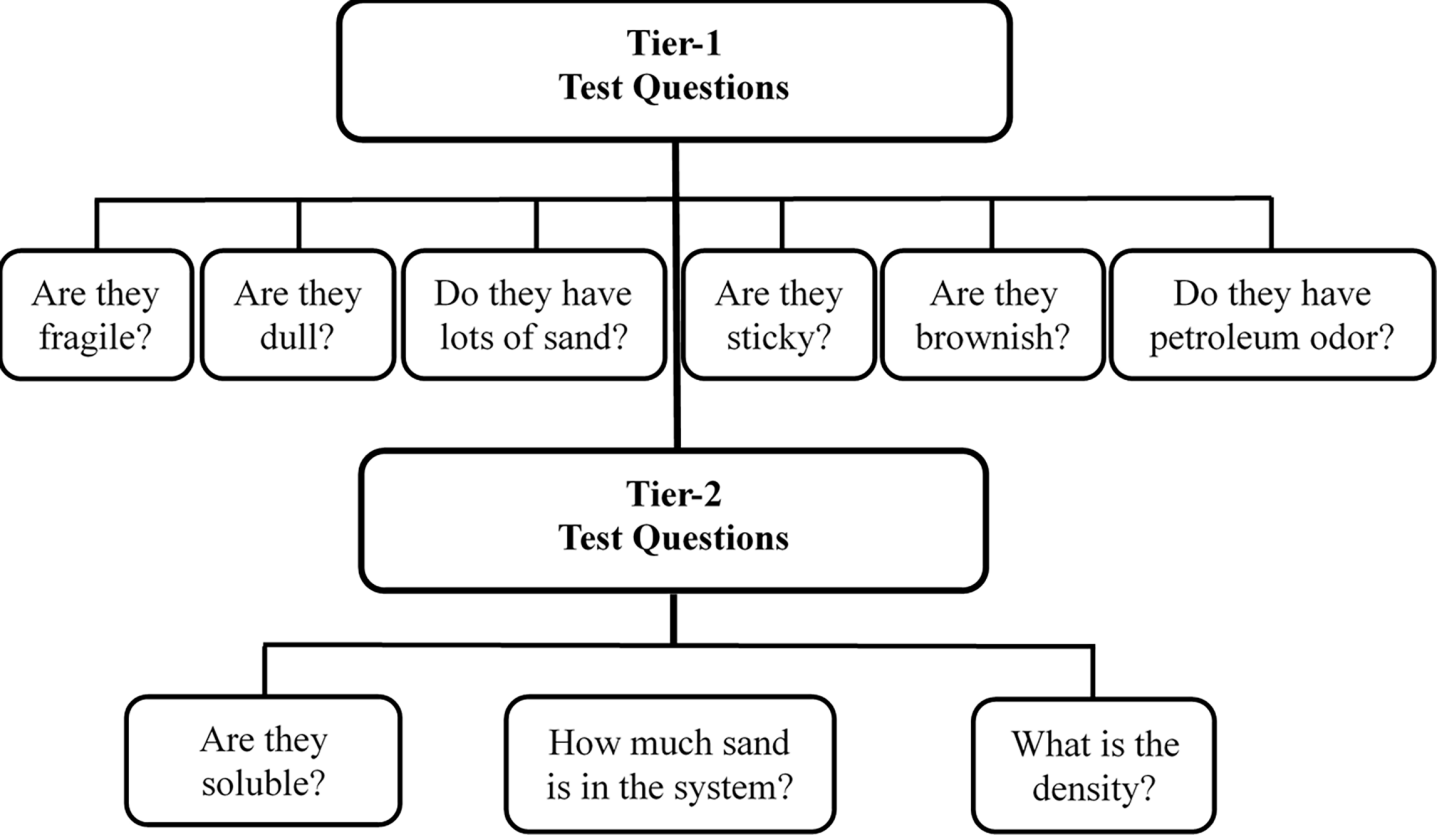
Details of proposed field testing protocol for identifying DWH oil spill residues.

Fig 2 summarizes the details of the proposed field testing protocol, which is a two-tier procedure that uses multiple physical characterization methods. As shown in the figure, the protocol includes a set of simple screening tests that can be conducted at any field site (identified as Tier-1 tests), and a set of confirmation tests that can be conducted in a temporary field laboratory (identified as Tier-2 tests). The Tier-1 tests can be conducted by any non-technical personal by following a simple set of directions that can be summarized in a two-page pamphlet. The Tier-2 tests require basic chemistry training and a temporary field laboratory. Since Tier-2 tests are confirmatory tests they can be done later, on a limited number of sub samples. In addition to Tier-1 and Tier-2 tests, we have also completed a series of advanced fingerprinting tests (identified as Tier-3 tests). The Tier-3 tests are not a part of the proposed field protocol; they were completed in this study to verify the results of the protocol. In routine field projects, the Tier-3 tests should only be occasionally performed to verify the findings.

#### 2.2.1 Tier-1 field testing methods

During field surveys, the oil spill residues can either be directly picked up by hand from dry beaches, or can be collected using a crab net from the swash zone [4]. The Tier-1 screening tests were designed based on the results from previously published studies which have demonstrated that the DWH oil spill residues found along sandy GOM beaches are fragile, dull, sticky, brownish and petroleum-smelling agglomerates that contain considerable amount of sand content [3–5,11,23]. As part of this screening procedure, six screening tests should be completed to answer the following set of yes or no questions:

1. Strength test: Are the samples fragile (as opposed to being hard and rubbery)? The objective is to test whether the samples can be easily broken apart into sand-grain sized particles.
2. Shine test: Are they dull? (typical DWH residues will be dull, whereas the non-DWH tar balls will be shiny, especially when they are broken apart)
3. Sand test: Do they have considerable amount of sand (as opposed to little or no sand)?
4. Stickiness test: Are they sticky when squeezed between fingers? When DWH oil spill residues are squeezed they will leave sticky residues.
5. Color test: Do they look brownish when broken apart? Typical DWH residues break into brownish aggregates (as opposed to dark black solids).
6. Odor test: Do the residue have a strong petroleum odor (as opposed to little or no petroleum odor)?

If the answers to all of the above questions are yes, it is likely that the residues have originated from the DWH oil spill.

#### 2.2.2 Tier-2 field testing methods

The Tier-2 tests are confirmatory tests that simply add further evidence to support Tier-1 findings. These tests require a makeshift laboratory that can be setup in a local public facility or in a temporary field facility. As part of Tier-2 screening, we propose to conduct the following two confirmatory tests:

1) Solvent test: Under field conditions, at times, it is difficult to distinguish certain petroleum residues from other marine debris. However, it has been well established that all types of petroleum residues are soluble in an organic solvent. The objective of this solvent test is to examine whether the sample will dissolve in an organic solvent (such as hexane, dichloromethane or gasoline). Commercial gasoline can be used if the test needs to be done in a field laboratory. Preliminary experiments completed in our laboratory have shown that DWH oil spill residues are fully soluble in commercial gasoline. If the solvent test was completed in a chemical laboratory, it is preferable to use dichloromethane since it can easily dissolve DWH residues as well as other highly weathered tar balls. It is important to note that the primary objective of the solubility test is to differentiate petroleum residues from other dark looking marine debris. A positive solvent test provides a conclusive evidence that the sample is indeed a petroleum residue. Furthermore, the DWH oil spill samples typically would dissolve in an organic solvent to yield a characteristic brownish solution, and also leave considerable amount of inorganic debris, such as sand and crushed shell fragments, at the bottom. These observations add further evidence that the sample could be of DWH origin.

2) Sand-content/ density test: The objective of this test is to evaluate sand and oil contents of the sample and use them to compute the relative density of the residue. The OSAT-2 [25] study has shown that DWH oil spill residues (both SOM and SRB/SOA samples) will have very high sand content ranging from 83.2% to 95.8% of sand by mass. Other previously published studies have also shown that the sand content in DWH residues collected from the Alabama beaches can range from 75% to 90% [5,11].

In order to estimate the amount of sand and shell fragments, the sample should be first homogenized by squeezing between fingers, and the aggregates should be gently dried using a paper towel. About 2 to 3 grams of the homogenized sample should be weighed using an analytical balance and transferred into a glass vial. The sample should then be sequentially washed using about 5 mL of organic solvent for at least four times. The dissolved oil should be decanted and discarded after each wash. The solids remaining at the bottom (DWH residues will have considerable amount of sand or shell fragments) should be first dried using a paper towel and then air-dried for a few minutes (to let all the residual solvents evaporate). The dried sample should be weighed to compute the sand fraction (f_sand_) and oil fraction (f_oil_) using the following formulae: f_oil_ = (wt. of original sample–wt. of sand)/wt. of original sample; and f_sand_ = 1 –f_oil_.

Density is an important physical parameter that can be used to differentiate DWH residues from traditional tar balls. Since DWH residues are a mixture of oil and sand (formed after the floating mousse sank mixing with sediments or after the beached mouse was buried under sand [15]), their relative density should always be higher than 1. According to a USGS study, the average relative density of DWH residues should be around 2 [26]. Traditional tar balls, in contrast, are mostly of pelagic origin (formed when the oil was floating over the ocean), and hence their relative density will be less than 1. In some cases, even traditional tar balls can sink after collecting little bit of sand on its surface. However, since most of these tar balls are made of hard and rubbery material, the sand particles cannot penetrate deep into the core and hence sand accumulation will be mostly limited to the surface, and therefore the net relative density can only be slightly higher than 1. Balkas et al. [27] reported that the relative density of the traditional floating and/or sunken tar balls could range from 0.80 to 1.25.

The relative density of an oil spill residue can be computed from the oil and sand values using the formula [26]: ρ = (f_oil_/0.9 + f_sand_/2.65)^-1^. This formula assumes that these residues are two-phase systems (oil/sand), and neither water nor air will be trapped within the sample. This is a reasonable approximation for most traditional tar balls. However, our observations have indicated that large DWH SRB/SOAs found along dry beaches can trap some air and hence their effective density can be slightly lower than the value estimated using the above formula.

#### 2.2.3 Tier-3 laboratory verification methods

Tier-3 uses several advanced laboratory testing methods that are primarily used here to validate the findings of the field protocol. All organic solvents used in this study were of analytical or higher grade. The organic solvents, silica gel (60–200 μm) and anhydrous sodium sulfate were purchased from VWR International (Suwanee, GA). A standard PAH mixture with 27 PAHs (naphthalene, 1-methylnaphthalene, 2-methylnaphthalene, 2,6-dimethylnaphthalene, 2,3,5-trimethylnaphthalene, biphenyl, acenaphthylene, acenaphthene, fluorene, phenanthrene, 1-methylphenanthrene, anthracene, dibenzothiophene, fluoranthene, pyrene, benzo(a)anthracene, chrysene, benzo(b)fluoranthene, benzo(j)fluoranthene, benzo(k)fluoranthene, benzo(e)pyrene, benzo(a)pyrene, perylene, dibenz(a,c)anthracene, dibenz(a,h)anthracene, indeno(1,2,3,-cd)pyrene and benzo(ghi)perylene) was purchased from Agilent Technologies (Wilmington, DE). The PAH surrogate standards (SS) consisting of naphthalene-*d*_*8*_, acenaphthene-*d*_*10*_, phenanthrene-*d*_*10*_ and benzo(a) pyrene-*d*_*12*_ were purchased from Ultra Scientific Analytical Solutions (North Kingstown, RI). Internal standard (IS) *p*-terphenyl-*d*_*14*_ (purity 98.5%) was purchased from AccuStandard (New Haven, CT).

The silica gel was activated according to a well-established protocol [28]. Column chromatographic fractionation step was conducted following published methods [5,28,29]. Briefly, a glass column (250 mm × 10 mm) with a Teflon stopcock was plugged with deactivated glass wool at the bottom and packed with 3 g of activated silica gel, and topped with 1 g of anhydrous sodium sulfate. The column was conditioned using 20 mL of hexane and the eluent was discarded. Sample containing about 25 mg of residual oil was weighed in a 12 mL vial and was dissolved using 1 mL of hexane. The solution was then spiked with 20 μL of 50 μg/mL four surrogate standards. The mixture was transferred to the column, and the vial was sequentially washed with 1 mL of hexane for twice; contents from sequential washes were transferred to the column. About 12 mL of hexane was added to the column to elute aliphatic hydrocarbon fractions, and this hexane fraction was labeled as F1. Then 15 mL of hexane: dichloromethane (50%, v/v) solvent mixture was used to elute the aromatic hydrocarbon fraction, and this fraction was labeled as F2. The F1 and F2 fractions were concentrated under a gentle stream of nitrogen and required amount of solvent was added to adjust the final volume to 10 mL. Exactly 1 mL of the adjusted F2 sample was spiked with internal standard of *p*-terphenyl-*d*_*14*_, prior to chemical analysis. All the samples were prepared in duplicate and analyzed in triplicate.

Agilent Gas Chromatograph coupled to an Agilent Mass Spectrometer was used to identify all the chemical compounds. Agilent’s Mass Hunter software was used to analyze the data. Chromatographic separation of various PAH compounds was achieved using a J&W DB-EUPAH (Agilent Technologies) column (20 m × 180 μm × 0.14 μm).

F1 fraction analysis was accomplished using a GC/MS single ion monitoring (SIM) mode. The hopanes and steranes fingerprints were observed at the target ion m/z values of 191 and 217, respectively. The electron ionization (EI) source temperature was set at 280°C with the carrier gas of Helium. The initial GC oven temperature of 50°C (0.5 min hold) was ramped to 310°C for 15 min at 6°C/min, resulting in total run time of 58.8 min. Diagnostic ratios (DRs) were estimated by computing various peak areas.

The F2 fraction was used to analyze the five groups of parent PAHs (naphthalene, phenanthrene, dibenzothiophene, fluorine and chrysene) and their alkylated homologs using a SIM method. The EI source temperature was set at 350°C. The inlet temperature was set at 300°C. The initial GC oven temperature of 50°C (1 min hold) was ramped to 300°C for 12 min at 5°C/min, resulting in total run time of 63 min. Commercially available PAH standards for naphthalene, 2-methylnaphthalene, 2,6-dimethylnaphthalene, 2,3,5-trimethylnaphthalene, phenanthrene, 1-methylphenanthrene, dibenzothiophene, fluorine and chrysene were used for identifying and quantifying the above five groups of compounds.

The calibration curves of PAHs used seven concentration levels: 5, 10, 20, 50, 100, 200, 500 ng/mL. All the calibration curves were found to be linear within the target concentration range. Since several alkylated PAHs standards are not commercially available, a semi-quantitative method was employed by using a relative response factor (RRF) to estimate alkylated PAH concentrations [5,28]. Specifically, C_0_-naphthalene was quantified by using naphthalene response; C_1_-naphthalenes by 2-methylnaphthalene; C_2_-naphthalenes by 2,6-dimethylnaphthalene; and C_3_- and C_4_-naphthalenes by 2,3,5-trimethylnaphthalene; C_0_-phenanthrene was quantified by phenanthrene; and C_1_- to C_4_-phenanthrenes by 1-methylphenanthrene. C_0_- to C_3_-dibenzothiophenes were quantified by dibenzothiophene; C_0_- to C_3_-fluorenes were quantified by fluorine, and C_0_- to C_4_-chrysenes were quantified by chrysene.

The F2 fraction of each sample was spiked with the internal standard *p*-terphenyl-*d*_*14*_ to compensate for any instrument variations. Four surrogate standards (naphthalene-*d*_*8*_, acenaphthene-*d*_*10*_, phenanthrene-*d*_*10*_ and benzo(a)pyrene-*d*_*12*_) were used to quantify recovery levels. The recovery levels of the four surrogates ranged from 72–91%, 69–95%, 70–97% and 85–131%, respectively. The limit of detection (LOD) and limit of quantitation (LOQ) for the PAH characterization method has been previously established as 0.20 to 3.65 ng/mL and 0.24 to 4.32 ng/mL, respectively [5].

## 3. Results and discussion

### 3.1 Tier-1 test results

**Fig 3:**
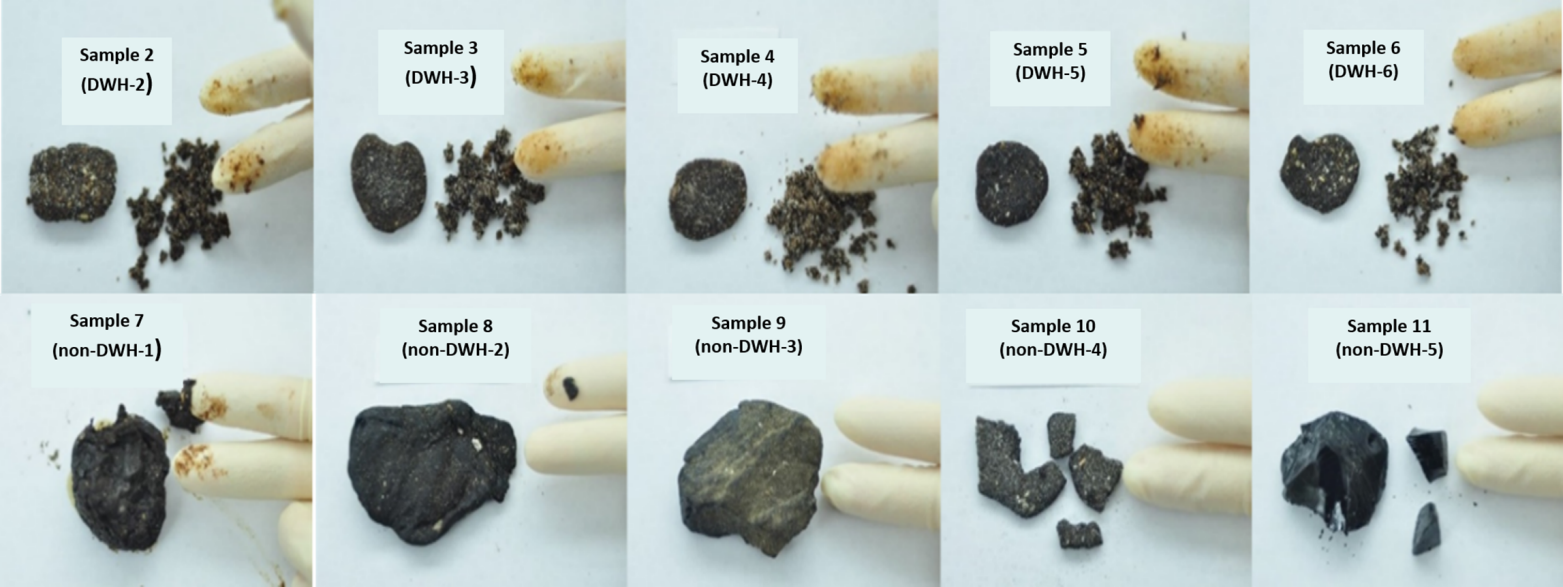
Tier-1 test results for the field samples.

Fig 3 shows digital photographs taken while conducting the Tier-1 tests for all the samples, except for Sample 1, a known DWH reference sample [23]. As shown in Fig 3, Samples 2 to 6 were all brownish samples with little or no shine. Samples 1 to 6 matched all the physical characteristics of DWH residues: they were all fragile, can be easily broken apart into fine fragments, contained large amounts of sand, and they left a sticky brownish stain on the finger. Samples 7 to 11 did not match one or more of the DWH residue physical characteristics. Specifically, Samples 7 to 11 were all dark (blackish), hard solid masses and hence were deemed as likely non-DWH residues. Some of these samples did have one or two DWH residue characteristics. For example, Sample 7 was sticky and soft and had a strong petroleum odor; Sample 8 was dull looking and had some petroleum odor; Sample 9 was dull looking; and Sample 10 had plenty of sand. Sample 11 was the only sample that failed to yield positive results to all six screening tests.

The results of all six Tier-1 screening tests completed for all 11 test samples are summarized in Table 2. The last column in this table indicates the preliminary conclusion that identifies the sample either as a DWH sample or as a non-DWH sample. In this protocol, a sample will be identified as a DWH sample if and only if all six screening test results are positive. If a sample yielded negative result even for one of the Tier-1 tests then it will be designated as a non-DWH sample. As shown in the table, Samples 1 to 6 yielded positive results for all six screening tests and hence were identified as DWH samples. Samples 7 to 11 did not yield positive result to one or more of the screening tests and hence were identified as non-DWH samples. Although, some of samples were already known to be non-DWH samples (for example, the Goa sample that came from India). In this study, we identified a sample as a non-DWH sample if and only if it failed to yield a positive answer to one or more of the Tier-1 screening questions. In addition to above tests, we also conducted Tier-1 screening tests to rapidly screen all 693 residues collected from Alabama’s beaches during our November 2015 survey (samples shown in Fig 1). Except for one sample (which was used as our test sample 10), all other 692 samples tested positive and had all six DWH oil spill residue characteristics.

**Table 2:** Summary of Tier-1 screening test results for all the field samples.

Sample name	Field screening of physical properties						Partial conclusion
	Fragile	Dull	Contain lot of sand	Sticky	Brownish	Petroleum odor	
Sample 1	Yes	Yes	Yes	Yes	Yes	Yes	DWH-1
Sample 2	Yes	Yes	Yes	Yes	Yes	Yes	DWH-2
Sample 3	Yes	Yes	Yes	Yes	Yes	Yes	DWH-3
Sample 4	Yes	Yes	Yes	Yes	Yes	Yes	DWH-4
Sample 5	Yes	Yes	Yes	Yes	Yes	Yes	DWH-5
Sample 6	Yes	Yes	Yes	Yes	Yes	Yes	DWH-6
Sample 7	No	No	No	Yes	No	Yes	Non-DWH-1
Sample 8	No	Yes	No	No	No	Yes	Non-DWH-2
Sample 9	No	Yes	No	No	No	No	Non-DWH-3
Sample 10	No	No	Yes	No	No	No	Non-DWH-4
Sample 11	No	No	No	No	No	No	Non-DWH-5

### 3.2 Tier-2 test results

#### 3.2.1 Solvent-test results

All eleven samples fully dissolved in the organic solvent (dichloromethane was used in this study). The solvent test indicated that all the samples are petroleum residues, and are not other marine debris. Samples 1 through 6 (DWH samples) dissolved to yield a brownish solution with considerable amount of white sand settling at the bottom of the vial. The non-DWH samples dissolved to yield a black solution with very little or no sediments at the bottom.

#### 3.2.2 Sand-content and density test results

Table 3 provides a summary of sand-content data collected for all 11 samples. The sand content of the DWH samples ranged from 77% to 87%, which is consistent with other published data [5,11,25]. The sand content values for most of the non-DWH samples (Sample 8, 9 and 11) were extremely low and ranged from 1% to 4%. However, two non-DWH sample did have relatively high amount of sand; Sample 7 (non-DWH-1) had 22% sand and Sample 10 (non-DWH-4) had 75% sand. The table also provides a summary of density values estimated for all 11 samples. The data show that the relative density values for DWH samples range from 1.83 to 2.12, which is within the expected range [26]. The relative density values for all the non-DWH samples, except for Sample 10 (non-DWH-4), were around 1, which is also consistent with literature data [27]. The Sample 10 was a clear outlier and had very high sand content and density values; however as shown in Table 2, this sample failed all other screening tests and hence it cannot be a DWH residue.

**Table 3:** Summary of Tier-2 test results.

Sample name	Solubility in DCM	Sand content	Relative density
Sample 1(DWH-1)	Soluble	0.87	2.12
Sample 2(DWH-2)	Soluble	0.83	2.00
Sample 3(DWH-3)	Soluble	0.85	2.04
Sample 4(DWH-4)	Soluble	0.80	1.91
Sample 5(DWH-5)	Soluble	0.82	1.95
Sample 6(DWH-6)	Soluble	0.77	1.83
Sample 7(non-DWH-1)	Soluble	0.22	1.05
Sample 8(non-DWH-2)	Soluble	0.04	0.92
Sample 9(non-DWH-3)	Soluble	0.01	0.90
Sample 10(non-DWH-4)	Soluble	0.75	1.78
Sample 11(non-DWH-5)	Soluble	0.01	0.90

### 3.3 Verification of the proposed field test results using Tier-3 chemical characterization datasets

#### 3.3.1 Results of hopane and sterane fingerprints

Source specific biomarkers such as hopane and sterane are widely used for forensic fingerprinting of oil spill residues. Biomarker compounds can provide important data for identifying the origin of the oil, monitoring the degree of weathering, and for quantifying biodegradation processes occurring under different environmental conditions [30,31]. Crude oils can be differentiated based on the relative distribution of different types of hopanes and steranes, and also the concentration levels of various other chemicals [18,20,21]. In this study we used hopanes and steranes to verify the findings of the proposed protocol that only Samples 1 to 6 should have originated from the DWH oil spill.

**Fig 4:**
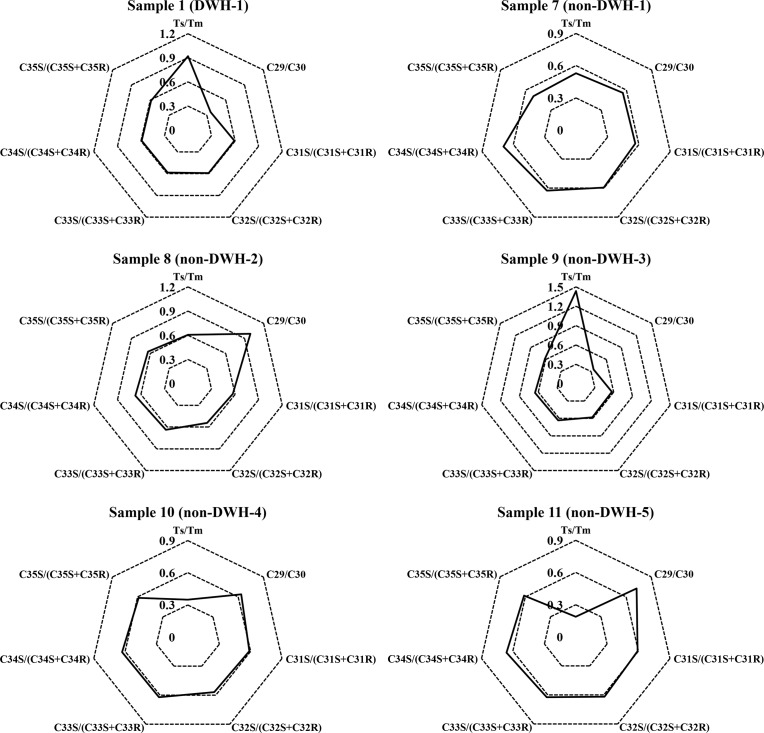
Radar plots of hopane diagnostic ratios of the field samples.

The diagnostic ratios (DRs) of various hopanes levels in all 11 test samples are listed in Table 4. These data show that seven different hopane DRs for Samples 2 to 6 are similar to the values estimated for Sample 1, a reference sample which has already been tested to match the fingerprint of DWH source crude [23]. These data show that the Samples 2 to 6 must have originated from the DWH oil. On the other hand, several of the DRs of non-DWH samples, especially the values of Ts/Tm and C_29_/C_30_, are significantly different from DWH samples indicating that these residues must have originated from sources other than the DWH crude. The ratios Ts/Tm and C29/C30, are some of the most reliable DRs that have been widely used for identifying petroleum sources [6,32,33]. When compared to Tm, Ts is more stable and less susceptible to degradation during sedimentation and diagenesis and therefore the ratio of Ts/Tm is an indicator of oil maturity [34]. Larger Ts/Tm indicates the higher maturity level of the crude oil. Similarly, C_29_ and C_30_ are another important set of thermodynamically stable hopanes that are resistant to weathering even under extreme conditions, and hence they are widely used for source identification [33]. Also, C_29_ and C_30_ are typically the most abundant hopanes in crude oils and therefore the ratio of C_29_/C_30_ can be estimated with a higher level of certainty [32]. The other five ratios are similar for all the 11 samples (see Table 4), indicating that these ratios are not good indicators for differentiating these oils. All seven different DRs of the five non-DWH samples are summarized in the form of radar plots and are then compared against the Sample 1 radar plot in Fig 4. These figures futher show that all the non-DWH samples must have originated from different types of sources.

**Table 4:** The diagnostic ratios (DRs) of hopanes for all the field samples.

Sample name	Ts/Tm	C29/C30	C31S/C31 (S+R)	C32S/C32 (S+R)	C33S/C33 (S+R)	C34S/C34 (S+R)	C35S/C35 (S+R)
Sample 1	0.92	0.36	0.59	0.59	0.58	0.59	0.59
Sample 2	0.94	0.37	0.59	0.59	0.59	0.61	0.60
Sample 3	0.93	0.37	0.59	0.60	0.59	0.60	0.58
Sample 4	0.93	0.38	0.59	0.59	0.60	0.60	0.59
Sample 5	0.93	0.36	0.59	0.60	0.59	0.59	0.58
Sample 6	0.93	0.36	0.60	0.60	0.59	0.60	0.59
Sample 7	0.53	0.56	0.57	0.60	0.63	0.69	0.51
Sample 8	0.61	0.99	0.57	0.54	0.64	0.67	0.64
Sample 9	1.44	0.35	0.57	0.58	0.63	0.65	0.62
Sample 10	0.35	0.64	0.59	0.57	0.62	0.63	0.59
Sample 11	0.19	0.73	0.59	0.62	0.62	0.66	0.62

**Fig 5:**
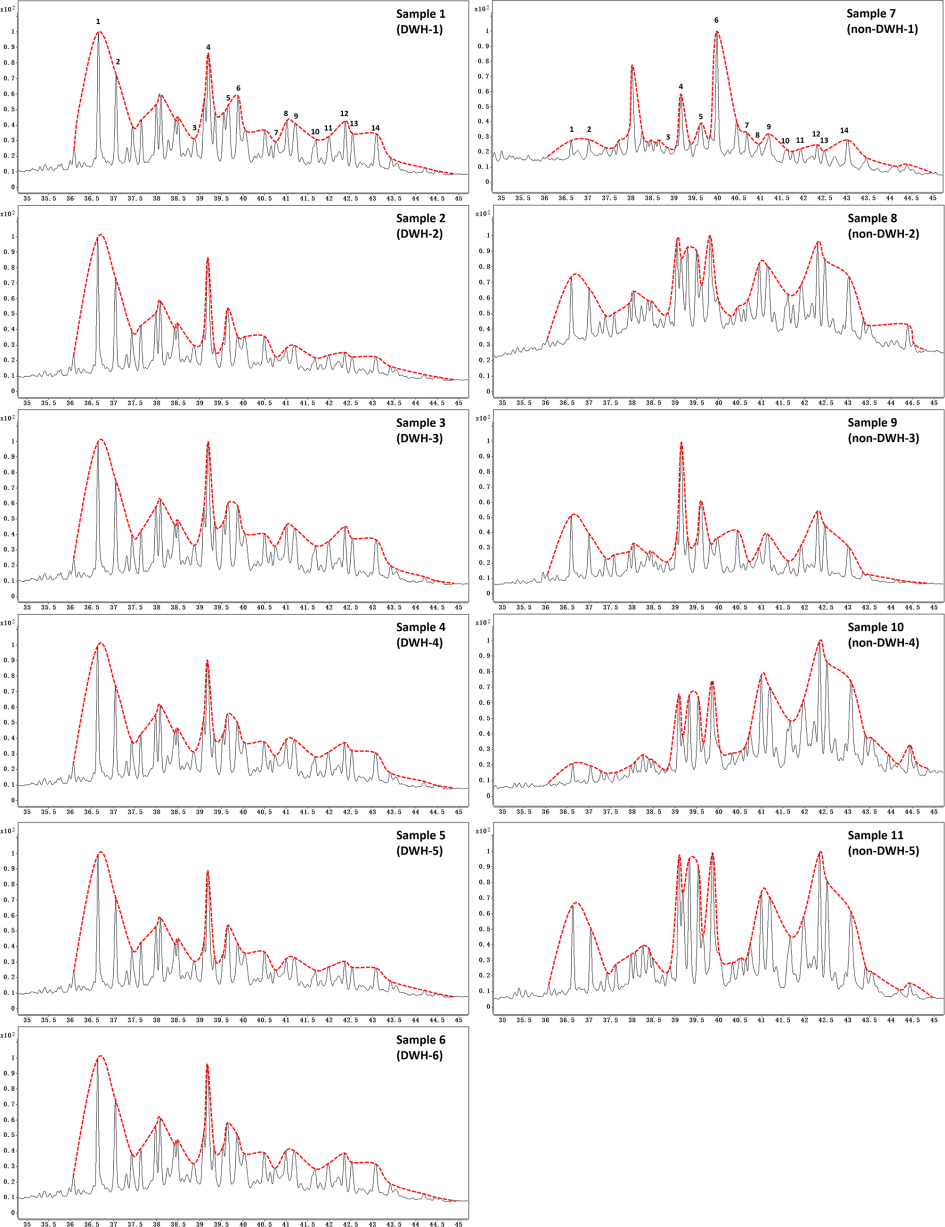
Mountain plots of sterane chromatograms (m/z = 217) for all eleven samples. Data for DWH residues are on the right and non-DWH samples are in the left. [Peak 1: DiaC27βα(S), 2: DiaC27βα(R), 3: C27ααα(S), 4: αββ(R), 5: C27αββ(S), 6: C27ααα(R), 7: C28ααα(S), 8: C28αββ(R), 9: C28αββ(S), 10: C28ααα(R), 11: C29ααα(S), 12: C29αββ(R), 13: C29αββ(S), 14: C29ααα(R)].

Similar to hopane, the relative distribution of various sterane compounds in a crude oil can be used to identify the oil source. Fig 5 shows the sterane chromatograms of all 11 samples presented in the form of mountain plots, which was developed by connecting various major peaks in sterane chromatograms. The figure shows that all DWH sample have similar looking mountain-peak pattern, whereas the non-DWH oils have mountain-peak patterns that are different from the DWH oil pattern. These data once again confirm that Samples 1 to 6 are the only samples that must have originated from the DWH crude.

#### 3.3.2 Analysis of PAH data

**Fig 6:**
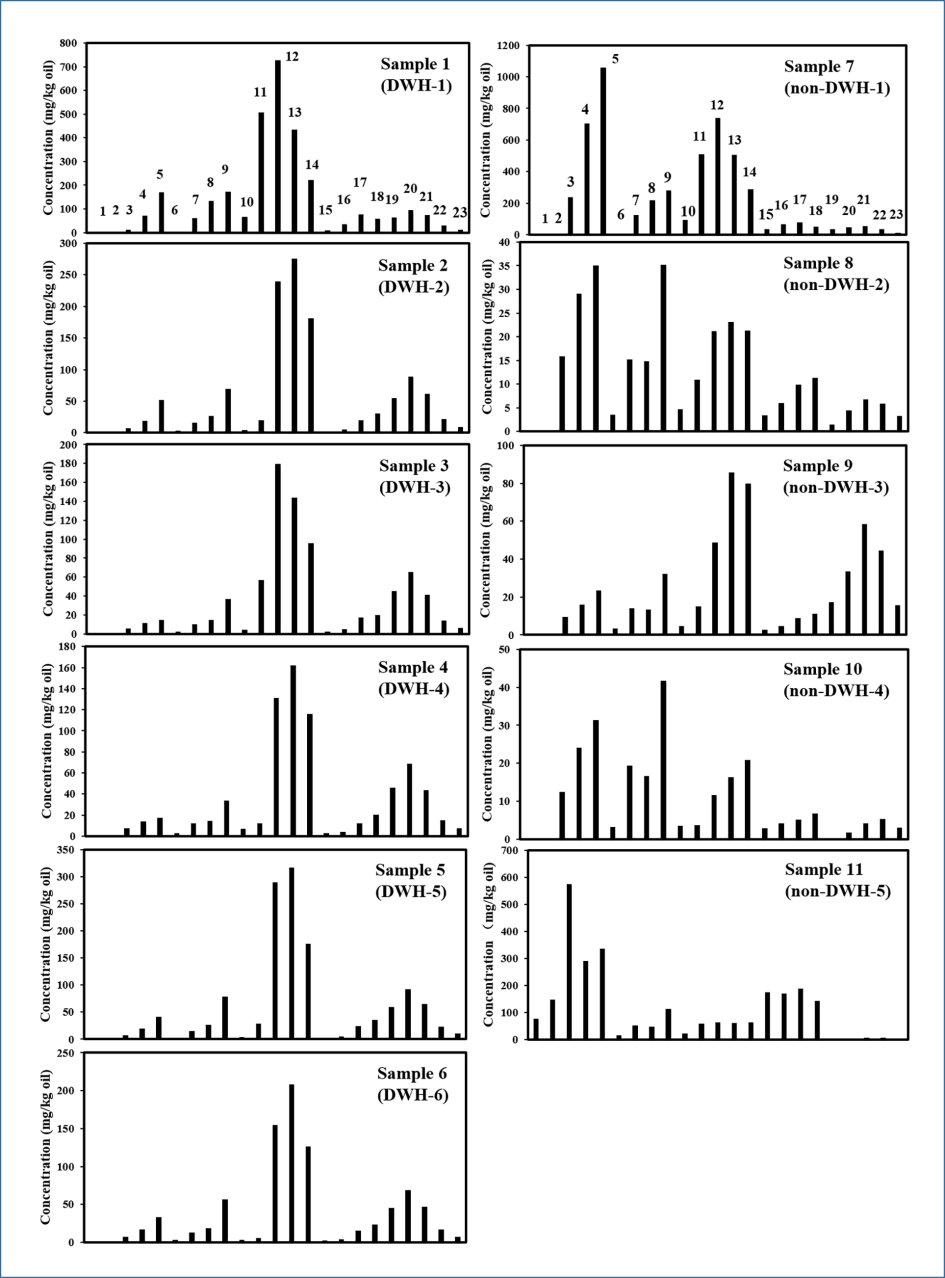
Comparison of PAH concentration levels of five important groups of PAHs and their alkylated homologs in the field samples. [1: C_0_-naphthalene, 2: C_1_-naphthalenes, 3: C_2_-naphthalenes, 4: C_3_-naphthalenes, 5: C_4_-naphthalenes, 6: C_0_-fluorene, 7: C_1_-fluorenes, 8: C_2_-fluorenes, 9: C_3_-fluorenes, 10: C_0_-phenanthrene, 11: C_1_-phenanthrenes, 12: C_2_-phenanthrenes, 13: C_3_-phenanthrenes, 14: C_4_-phenanthrenes, 15: C_0_-dibenzothiophene, 16: C_1_-dibenzothiophenes, 17: C_2_-dibenzothiophenes, 18: C_3_-dibenzothiophenes, 19: C_0_-chrysene, 20: C_1_-chrysenes, 21: C_2_-chrysenes, 22: C_3_-chrysenes, 23: C_4_-chrysenes].

PAHs are an important group of toxic compounds present in oil spill residues, and several of them are listed in USEPA’s priority pollutant list [35]. Investigators have used the relative distribution of different types of PAHs to distinguish the oil spill source [18,29,36]. The concentration distribution of 5 major types of most abundant PAHs (naphthalenes, fluorenes, phenanthrenes, dibenzothiophenes and chrysenes) and their alkylated PAH homologs in all eleven oil spill samples were estimated, and results are summarized in Table 5. Fig 6 shows the relative distribution of these five major groups of PAHs in all eleven samples. These data show that the six DWH samples have similar distribution patterns, whereas the non-DWH samples have different patterns. The concentration data summarized in the table indicate that among the 5 groups of major PAHs, phenanthrene and its alkylated homologs are the most dominant compounds in all DWH samples, ranging between 44% to 63% of total PAHs. The percentage of phenanthrenes in Samples 7 through 11 (non-DWH samples) are: 40%, 24%, 39%, 20% and 10%, respectively; overall, these values are relatively lower when compared to DWH sample values.

**Table 5:** Concentrations of PAHs measured in all the field samples.

Compound	Sample 1	Sample 2	Sample 3	Sample 4	Sample 5	Sample 6	Sample 7	Sample 8	Sample 9	Sample 10	Sample 11
C_0_-naphthalene	DL	DL	DL	DL	DL	DL	DL	DL	DL	DL	77.8
C_1_-naphthalenes	DL	DL	DL	DL	DL	DL	6.4	DL	DL	DL	149.2
C_2_-naphthalenes	11.3	7.1	5.9	7.5	6.9	7.7	235.7	15.8	9.6	12.5	575.4
C_3_-naphthalenes	72.6	19.0	11.4	14.0	19.9	16.9	706.0	29.1	16.0	24.2	291.4
C_4_-naphthalenes	169.0	51.4	14.6	17.4	40.9	33.6	1056.6	35.0	23.6	31.4	336.4
C_0_-phenanthrene	65.3	4.1	4.3	6.9	3.7	3.5	92.8	4.7	4.5	3.5	23.6
C_1_-phenanthrenes	506.0	19.7	56.9	12.3	28.6	5.6	508.5	10.9	15.0	3.6	60.2
C_2_-phenanthrene	728.2	239.9	179.2	130.9	289.5	154.6	738.2	21.2	48.8	11.7	64.0
C_3_-phenanthrene	435.3	275.8	144.1	162.3	316.5	208.6	504.1	23.1	85.7	16.2	61.2
C_4_-phenanthrene	222.5	181.0	95.6	115.8	176.3	126.4	287.8	21.3	80.0	20.8	64.4
C_0_-dibenzothiophene	7.9	2.8	2.6	2.9	2.5	2.7	34.4	3.4	2.9	2.8	176.4
C_1_-dibenzothiophenes	34.0	4.8	5.2	4.1	4.7	4.1	66.6	6.0	4.5	4.1	171
C_2_-dibenzothiophenes	76.4	20.0	17.2	12.0	24.4	15.1	79.5	9.9	8.7	5.1	188.6
C_3_-dibenzothiophenes	58.6	30.4	20.0	20.1	35.3	23.7	52.0	11.4	11.2	6.7	142.8
C_0_-fluorene	5.0	2.9	2.8	3.0	2.7	2.9	9.5	3.5	3.2	3.2	16.4
C_1_-fluorenes	59.9	15.6	10.4	12.2	15.6	12.7	123.9	15.3	14.2	19.3	52.0
C_2_-fluorenes	134.3	26.2	15.1	14.6	27.0	18.5	218.9	14.8	13.3	16.6	48.0
C_3_-fluorenes	171.5	69.9	36.7	34.0	78.8	56.9	280.1	35.1	32.2	41.8	114.2
C_0_-chrysenen	62.6	54.7	45.2	46.2	59.8	45.6	36.8	1.5	17.3	0.1	1.2
C_1_-chrysenens	95.9	89.1	65.7	68.9	92.5	68.5	45.8	4.4	33.5	1.7	2.6
C_2_-chrysenens	73.4	61.7	41.7	43.6	65.4	47.1	55.9	6.8	58.5	4.1	6.8
C_3_-chrysenens	30.6	22.2	14.1	14.9	23.6	17.2	35.9	5.8	44.5	5.2	6.6
C_4_-chrysenens	12.0	8.7	6.5	7.6	10.1	7.2	13.7	3.3	15.8	3.0	2.6

## 4. Summary and conclusions

In this study, we propose a two-tier field testing protocol for monitoring DWH oil spill residues found along sandy GOM beaches. The proposed method uses six physical characterization tests to differentiate DWH residues from other types of petroleum residues. The field protocol was tested using eleven samples collected from different sites. The goal was to use the protocol to test a working hypothesis that these eleven samples could have originated from the DWH oil. The Tier-1 analysis for Samples 1 to 6 yielded positive results for all the physical characterization tests, and hence supported the hypothesis that these samples must have originated from the DWH oil. The Tier-2 results indicated that Samples 1 to 6 will dissolve in an organic solvent, and their relative density values are close to 2. These results provided further confirmative evidence for supporting the hypothesis. The Samples 7 to 11, on the other hand, yielded negative results for one or more of the Tier-1 tests. Therefore, we reject the working hypothesis and conclude that Samples 7 to 11 did not originate from the DWH oil spill. These conclusions were independently verified by completing a detailed set of advanced chemical characterization tests on all eleven samples. The chemical characterization results verified the conclusions derived using the proposed field testing protocol.

As pointed out in previous studies [3,4], the sandy beaches located along the GOM shoreline, which were severely impacted by the DWH spill, have been slowly recovering over the past seven years. However, some of these beaches are continuing to be impacted by the residues trapped in the nearshore environment. For example, the DWH mousse submerged near the Alabama shoreline still continue to deposit large amount of oil spill residues along Alabama’s amenity beaches; this has increased the background oil level in this region by about four orders of magnitude [3]. Furthermore, these DWH residues contain considerable amounts of recalcitrant toxic PAHs that are weathering rather slowly [5,11,23]. Therefore, it is important that we continue to monitor the fate of DWH oil spill residues and fully understand their long-term environmental impacts. Unfortunately, the local city/municipal offices or environmental management agencies have very little resources to employ highly trained laboratory staff to conduct these long-term beach monitoring surveys. If and when possible, they instead use field workers and/or volunteers to conduct these surveys. Also, the amount of oil deposited along these beaches could have large variations, and the amount can increase substantially after a major storm event [4]. For example, a few days after the landfall of Tropical Storm Isaac (which crossed the GOM shoreline on August 29, 2012) our team collected large amount of (about 11 kg) residues from the Alabama shoreline [4]. In situations like these, it is cost-prohibitive to conduct detailed laboratory characterization studies on all the field samples. Therefore, there is a need for a simple method that can be cost-effectively used by field workers to rapidly identify DWH oil spill residues. Our study addresses this need and provides a reliable, cost-effective and practical method for differentiating DWH residues from other types of petroleum residues.

## References

[1] McNutt MK, Camilli R, Crone TJ, Guthrie GD, Hsieh PA, Ryerson TB, et al (2012) Review of flow rate estimates of the Deepwater Horizon oil spill. Proceedings of the National Academy of Sciences of the United States of America 109: 20260–20267. doi: 10.1073/pnas.1112139108 22187459 10.1073/pnas.1112139108 PMC3528583

[2] Michel J, Owens EH, Zengel S, Graham A, Nixon Z, Allard T, et al (2013) Extent and degree of shoreline oiling: Deepwater Horizon oil spill, Gulf of Mexico, USA. Plos One 8: e65087 doi: 10.1371/journal.pone.0065087 23776444 10.1371/journal.pone.0065087 PMC3680451

[3] Clement TP, John GF, Yin F (2017) Chapter 16—Assessing the Increase in Background Oil–Contamination Levels Along Alabama's Beaches Resulting From the Deepwater Horizon Oil Spill In: Fingas M, editor. Oil Spill Science and Technology (Second Edition). Boston: Elsevier Inc. pp. 851–888.

[4] Hayworth JS, Clement TP, John GF, Yin F (2015) Fate of Deepwater Horizon oil in Alabama's beach system: Understanding physical evolution processes based on observational data. Mar Pollut Bull 90: 95–105. doi: 10.1016/j.marpolbul.2014.11.016 25496697 10.1016/j.marpolbul.2014.11.016

[5] Yin F, John GF, Hayworth JS, Clement TP (2015) Long-term monitoring data to describe the fate of polycyclic aromatic hydrocarbons in Deepwater Horizon oil submerged off Alabama's beaches. Science of the Total Environment 508: 46–56. doi: 10.1016/j.scitotenv.2014.10.105 25437952 10.1016/j.scitotenv.2014.10.105

[6] Aeppli C, Nelson RK, Radovic JR, Carmichael CA, Valentine DL, Reddy CM (2014) Recalcitrance and Degradation of Petroleum Biomarkers upon Abiotic and Biotic Natural Weathering of Deepwater Horizon Oil. Environmental Science & Technology 48: 6726–6734. 24831878 10.1021/es500825q

[7] Liu ZF, Liu JQ, Zhu QZ, Wu W (2012) The weathering of oil after the Deepwater Horizon oil spill: insights from the chemical composition of the oil from the sea surface, salt marshes and sediments. Environmental Research Letters 7.

[8] Urbano M, Elango V, Pardue JH (2013) Biogeochemical characterization of MC252 oil:sand aggregates on a coastal headland beach. Marine Pollution Bulletin 77: 183–191. doi: 10.1016/j.marpolbul.2013.10.006 24210008 10.1016/j.marpolbul.2013.10.006

[9] Kennicutt MC (2017) Oil and Gas Seeps in the Gulf of Mexico. Habitats and Biota of the Gulf of Mexico: Before the Deepwater Horizon Oil Spill: Springer pp. 275–358.

[10] MacDonald IR, Garcia-Pineda O, Beet A, Asl SD, Feng L, Graettinger G, et al (2015) Natural and unnatural oil slicks in the Gulf of Mexico. Journal of Geophysical Research-Oceans 120: 8364–8380. doi: 10.1002/2015JC011062 27774370 10.1002/2015JC011062 PMC5064732

[11] Mulabagal V, Yin F, John GF, Hayworth JS, Clement TP (2013) Chemical fingerprinting of petroleum biomarkers in Deepwater Horizon oil spill samples collected from Alabama shoreline. Mar Pollut Bull 70: 147–154. doi: 10.1016/j.marpolbul.2013.02.026 23523118 10.1016/j.marpolbul.2013.02.026

[12] NRC (2003) Oil in the Sea III:: Inputs, Fates, and Effects: national academies Press. 25057607

[13] Fingas M, Fieldhouse B (2004) Formation of water-in-oil emulsions and application to oil spill modelling. Journal of Hazardous Materials 107: 37–50. doi: 10.1016/j.jhazmat.2003.11.008 15036641 10.1016/j.jhazmat.2003.11.008

[14] Goodman R (2003) Tar balls: The end state. Spill Science & Technology Bulletin 8: 117–121.

[15] Gustitus SA, Clement TP (2018) Formation, Fate, and Impacts of Microscopic and Macroscopic Oil-Sediment Residues in Nearshore Marine Environments: A Critical Review. Reviews of Geophysics doi: 10.1002/2017RG000572.

[16] Stout SA (2016) Oil spill fingerprinting method for oily matrices used in the Deepwater Horizon NRDA. Environmental Forensics 17: 218–243.

[17] Suneel V, Vethamony P, Naik BG, Kumar KV, Sreenu L, Samiksha SV, et al (2014) Source Investigation of the Tar Balls Deposited along the Gujarat Coast, India, Using Chemical Fingerprinting and Transport Modeling Techniques. Environmental Science & Technology 48: 11343–11351. 25198506 10.1021/es5032213

[18] Wang ZD, Fingas M, Page DS (1999) Oil spill identification. Journal of Chromatography A 843: 369–411.

[19] Zakaria MP, Horinouchi A, Tsutsumi S, Takada H, Tanabe S, Ismail A (2000) Oil pollution in the Straits of Malacca, Malaysia: Application of molecular markers for source identification. Environmental Science & Technology 34: 1189–1196.

[20] Hostettler FD, Lorenson TD, Bekins BA (2013) Petroleum Fingerprinting with Organic Markers. Environmental Forensics 14: 262–277.

[21] Wang ZD, Stout SA, Fingas M (2006) Forensic fingerprinting of biomarkers for oil spill characterization and source identification. Environmental Forensics 7: 105–146.

[22] Wang Z, Stout S (2010) Oil spill environmental forensics: fingerprinting and source identification: Academic Press.

[23] John GF, Han YL, Clement TP (2016) Weathering patterns of polycyclic aromatic hydrocarbons contained in submerged Deepwater Horizon oil spill residues when re-exposed to sunlight. Science of the Total Environment 573: 189–202. doi: 10.1016/j.scitotenv.2016.08.059 27565528 10.1016/j.scitotenv.2016.08.059

[24] Suneel V, Vethamony P, Zakaria MP, Naik BG, Prasad KVSR (2013) Identification of sources of tar balls deposited along the Goa coast, India, using fingerprinting techniques. Mar Pollut Bull 70: 81–89. doi: 10.1016/j.marpolbul.2013.02.015 23522683 10.1016/j.marpolbul.2013.02.015

[25] OSAT (2011) Summary Report for Fate and Effects of Remnant Oil in the Beach Environment.

[26] Plant NG, Long JW, Dalyander PS, Thompson DM, Raabe EA (2013) Application of a hydrodynamic and sediment transport model for guidance of response efforts related to the Deepwater Horizon oil spill in the Northern Gulf of Mexico along the coast of Alabama and Florida. Reston, VA. 2012–1234 2012–1234. 60 p.

[27] Balkas TI, Salihoglu I, Gaines AF, Sunay M, Matthews J (1982) Characterization of Floating and Sinking Tar Balls in the Marine-Environment. Mar Pollut Bull 13: 202–205.

[28] Wang ZD, Fingas M, Li K (1994) Fractionation of a Light Crude-Oil and Identification and Quantitation of Aliphatic, Aromatic, and Biomarker Compounds by Gc-Fid and Gc-Ms .2. J Chromatogr Sci 32: 367–382.

[29] Yin F, Hayworth JS, Clement TP (2015) A Tale of Two Recent Spills-Comparison of 2014 Galveston Bay and 2010 Deepwater Horizon Oil Spill Residues. Plos One 10. 10.1371/journal.pone.0118098 PMC4340883 25714100

[30] Wang ZD, Fingas M (2003) Fate and identification of spilled oils and petroleum products in the environment by GC-MS and GC-FID. Energy Sources 25: 491–508.

[31] Fingas M (2016) Oil spill science and technology: Gulf professional publishing.

[32] Wang ZD, Fingas M, Sergy G (1994) Study of 22-Year-Old Arrow Oil Samples Using Biomarker Compounds by Gc/Ms. Environmental Science & Technology 28: 1733–1746. 22176377 10.1021/es00058a027

[33] Peters KE, Walters CC, Moldowan JM (2005) The biomarker guide: biomarkers and isotopes in the environment and human history: Cambridge University Press.

[34] Shen J (1984) Minimization of Interferences from Weathering Effects and Use of Biomarkers in Identification of Spilled Crude Oils by Gas-Chromatography Mass-Spectrometry. Analytical Chemistry 56: 214–217.

[35] USEPA (1993) Provisional guidance for quantitative risk assessment of polycyclic aromatic hydrocarbons. Washington, DC. EPA/600/R-693/089 p.

[36] Wang ZD, Fingas M, Sergy G (1995) Chemical Characterization of Crude-Oil Residues from an Arctic Beach by Gc/Ms and Gc/Fid. Environmental Science & Technology 29: 2622–2631. 22191964 10.1021/es00010a025

